# Upregulation of *Apol8* by Epothilone D facilitates the neuronal relay of transplanted NSCs in spinal cord injury

**DOI:** 10.1186/s13287-021-02375-w

**Published:** 2021-05-26

**Authors:** Weiwei Xue, Haipeng Zhang, Yongheng Fan, Zhifeng Xiao, Yannan Zhao, Weiyuan Liu, Bai Xu, Yanyun Yin, Bing Chen, Jiayin Li, Yi Cui, Ya Shi, Jianwu Dai

**Affiliations:** 1 State Key Laboratory of Molecular Developmental Biology, Institute of Genetics and Developmental Biology, Chinese Academy of Sciences, Beijing, 100101 China; 2 University of the Chinese Academy of Sciences, Beijing, 100190 China; 3 Reproductive and Genetic Center of National Research Institute for Family Planning, Beijing, 100081 China; 4 Key Laboratory for Nano-Bio Interface Research, Division of Nanobiomedicine, Suzhou Institute of Nano-Tech and Nano-Bionics, Chinese Academy of Sciences, Suzhou, 215123 China

**Keywords:** Epothilone D, Apol8, Neuronal differentiation, Neural stem cell, Spinal cord injury

## Abstract

**Background:**

Microtubule-stabilizing agents have been demonstrated to modulate axonal sprouting during neuronal disease. One such agent, Epothilone D, has been used to treat spinal cord injury (SCI) by promoting axonal sprouting at the lesion site after SCI. However, the role of Epothilone D in the differentiation of neural stem cells (NSCs) in SCI repair is unknown. In the present study, we mainly explored the effects and mechanisms of Epothilone D on the neuronal differentiation of NSCs and revealed a potential new SCI treatment.

**Methods:**

In vitro differentiation assays, western blotting, and quantitative real-time polymerase chain reaction were used to detect the effects of Epothilone D on NSC differentiation. Retrograde tracing using a pseudotyped rabies virus was then used to detect neuronal circuit construction. RNA sequencing (RNA-Seq) was valuable for exploring the target gene involved in the neuronal differentiation stimulated by Epothilone D. In addition, lentivirus-induced overexpression and RNA interference technology were applied to demonstrate the function of the target gene. Last, an *Apol8*-NSC-linear ordered collagen scaffold (LOCS) graft was prepared to treat a mouse model of SCI, and functional and electrophysiological evaluations were performed.

**Results:**

We first revealed that Epothilone D promoted the neuronal differentiation of cultured NSCs and facilitated neuronal relay formation in the injured site after SCI. Furthermore, the RNA-Seq results demonstrated that *Apol8* was upregulated during Epothilone D-induced neuronal relay formation. Lentivirus-mediated *Apol8* overexpression in NSCs (*Apol8*-NSCs) promoted NSC differentiation toward neurons, and an *Apol8* interference assay showed that *Apol8* had a role in promoting neuronal differentiation under the induction of Epothilone D. Last, *Apol8*-NSC transplantation with LOCS promoted the neuronal differentiation of transplanted NSCs in the lesion site as well as synapse formation, thus improving the motor function of mice with complete spinal cord transection.

**Conclusions:**

Epothilone D can promote the neuronal differentiation of NSCs by upregulating *Apol8*, which may provide a promising therapeutic target for SCI repair.

## Background

Spinal cord injury (SCI) is a serious condition of the central nervous system (CNS), and SCI repair is a great clinical challenge. After SCI, cell death, ischemia, excitotoxicity, edema, immune reaction, and other phenomena occur at the injured site, and a series of pathological reactions form an inhibitory microenvironment that blocks nerve regeneration [1, 2]. Myelin-associated inhibitors, including Nogo, myelin-associated glycoprotein, and oligodendrocyte myelin glycoprotein, inhibit CNS axon regeneration and sprouting [3]. After SCI, a large number of neurons are lost and axons gradually deteriorate [4]. SCI leads to permanent functional defects because of the loss of neurons and axons, while the spontaneous regeneration of neurons and axons is limited [5]. Functional connections between neurons are lost, nerve impulse transmission terminates, and a large number of astrocytes around the damaged area are activated, which forms a physical barrier or glial scar, in the damaged area, seriously hindering axonal regrowth and the reconstruction of new neural circuits [6].

Neural stem cells (NSCs) play an important role in the nervous system and have attracted much attention in SCI studies because of their ability to differentiate into neurons and glial cells [7, 8]. NSC transplantation is not only a simple replacement of lost nerve cells in the damaged area; NSCs can also secret nutritional factors, promote cell survival, and stimulate bridge formation between axons and the host to promote functional recovery [9–11]. NSC transplantation can compensate for the loss of neurons and can provide seed cells for clinical SCI treatment, which is considered to be a promising strategy for the treatment of severe SCI. However, many studies have shown that the vast majority of transplanted NSCs differentiate into astrocytes in the SCI inhibitory microenvironment but cannot differentiate into neurons [12–14]. Promoting neuronal differentiation of NSCs may be effective in SCI repair.

Many growth factors, antibodies, and chemicals can benefit the neuronal differentiation of NSCs. However, effective differentiation of NSCs has not been fully resolved [15]. NSC differentiation depends, in part, on cell cycle regulation, and cell fate can be determined by the distribution of genetic material through spindle microtubule stretching [16]. In recent years, some anticancer drugs that stabilize microtubules have been used in the treatment of SCI. Our recent study found that cetuximab, an anticancer drug, antagonized the SCI inhibitory microenvironment to promote neuronal differentiation of NSCs through blocking of the EGFR–ERK pathway [17]. In addition, the anticancer drug, paclitaxel, can promote the growth of neuronal axons by stabilizing microtubules. A low dose of paclitaxel can promote the neuronal differentiation of NSCs and inhibit their differentiation into astrocytes by stabilizing microtubules, which promotes the recovery of motor function after SCI in rodents [18–20]. However, paclitaxel administered after SCI can be toxic to cells and tends to diffuse, making it difficult to control the concentration of the drug. In recent years, Ruschel et al. have shown that Epothilone B stabilizes microtubules, can reduce scarring, and stimulates axonal regeneration, thereby restoring motor function after SCI [21, 22]. Epothilone D, a substitute for Epothilone B, has greater blood–brain barrier permeability and displays better bioavailability and effectiveness in rodent CNS disease models. Microtubule-stabilizing drugs are effective in the treatment of SCI and neurodegenerative diseases, such as Alzheimer’s disease [23]. Compared with paclitaxel, Epothilone D is simple in structure and is more conducive to chemical synthesis. It also has better water solubility and lower cytotoxicity and crosses the blood–brain barrier more easily [24]. Epothilone D can improve hind limb control in adult rats with contusion SCI, can reduce inhibitory fibrotic scar tissue in damaged areas, and can restore nerve innervation in the lumbar spine [25]. Epothilone D promotes axonal bud outburst and affects the cell cycle by inhibiting the transition of the G1 phase to the S phase [26]. Together, these findings indicate that drugs or small molecules that stabilize microtubules may play an important role in nerve regeneration for SCI repair.

In this study, we found that Epothilone D promoted *Apol8* expression and that *Apol8* overexpression can facilitate the neuronal differentiation of NSCs. Furthermore, *Apol8*-overexpressing NSCs transplanted into the injured site after SCI preferred to differentiate into neurons rather than astrocytes. In addition, the newly generated neurons were involved in the regeneration of synaptic structures, which enhanced the recovery of motor function after SCI.

## Methods

### Proliferation and differentiation of NSCs

The NSCs were obtained from the spinal cords of postnatal mice (within 12 h of birth) and cultured in Dulbecco’s modified Eagle’s medium/F12 containing glucose (30%), B27 supplement (2%, 17504-044; Gibco), epidermal growth factor (20 ng/mL), fibroblast growth factor (20 ng/mL), and heparin (1.83 μg/mL) at 37°C in 5% CO_2_. After 10 days, the NSC spheres were digested to single cells and were attached to plates for differentiation in Dulbecco’s modified Eagle’s medium/F12 containing B27 supplement (2%, 17504-044; Gibco), non-essential amino acids (1%, 11140-050; Gibco), sodium pyruvate (1%, 11360-070; Gibco), and penicillin-streptomycin (1%, 15140-122; Gibco).

### Immunofluorescence and histological analysis

Cells were fixed in 4% paraformaldehyde for 30 min and treated with 0.1% Triton X-100 for 15 min at room temperature (RT). Cells were then blocked in 5% bovine serum albumin (BSA) for 30 min before primary antibody addition. Cells were incubated with primary antibodies for 12 h at 4°C (beta-tubulin III [TUJ1] antibody: ab18207, rabbit, 1:500; microtubule-associated protein 2 [MAP2] antibody: ab5392, chicken, 1:500; glial fibrillary acidic protein [GFAP] antibody: ab7260, rabbit, 1:500; all Abcam). After washing three times in Dulbecco’s phosphate-buffered saline, Alexa Fluor-conjugated secondary antibodies were incubated at RT for 1 h. The nuclei were stained by Hoechst 33342 (DH164-1, 1:1000, Sigma-Aldrich).

The spinal cords were obtained after paraformaldehyde fixation, dehydrated with sucrose (20% and 30% sequentially), and then embedded in an optimal cutting temperature compound. The frozen tissue was sectioned at 12 μm, and the sections were incubated in 5% BSA for 1 h, and then with primary antibodies overnight at 4°C (green fluorescence protein [GFP] antibody: ab13970, chicken, 1:500; doublecortin [DCX] antibody: ab18723, rabbit, 1:500; TUJ1 antibody: ab18207, rabbit, 1:500; neuronal N [NeuN] antibody: ab177487, rabbit, 1:500; GFAP antibody: ab7260, rabbit, 1:500; red fluorescent protein [RFP] antibody: ab8135, rabbit, 1:500; all Abcam; synaptophysin [SYN] antibody: MAB329, mouse, 1:500, Millipore; postsynaptic density protein 95 [PSD95] antibody: MAB1596, mouse, 1:500, Millipore; neurofilaments [NF] antibody: N4142, rabbit, 1:500, Sigma). On the second day, the sections were incubated with Alexa Fluor-conjugated secondary antibodies (1:500, Invitrogen) at RT for 1 h. The nuclei were stained with Hoechst 33342 dye (1 mg/mL).

### Western blotting

Differentiated cells were scraped from the plate and were lysed in radioimmunoprecipitation assay buffer (WB-0071, China) for 20 min on ice. For each sample, 40 μg of protein was then separated by sodium dodecyl sulfate polyacrylamide gel electrophoresis. The membranes were incubated with primary antibodies diluted to 1:1000 for 12 h at 4°C (glyceraldehyde 3-phosphate dehydrogenase [GAPDH] antibody: TA-08, mouse, Santa Cruz; TUJ1 antibody: ab18207, rabbit; MAP2 antibody: ab32454, rabbit; GFAP antibody: ab7260, rabbit; all Abcam). After three washes, the membranes were incubated with horseradish peroxidase-conjugated secondary antibodies at 1:3000 dilution for 1 h at RT.

### RNA sequencing (RNA-Seq)

After SCI, mice were intraperitoneally injected with Epothilone D at 0.75 mg/kg for 5 days. Then, a 2-mm length of the spinal cord at the T8 injury site was obtained and sent to Shanghai Ouyi Biomedical Technology Co., Ltd. for RNA-Seq.

### Quantitative real-time polymerase chain reaction (qRT-PCR)

Total RNA of differentiated cells was extracted using TRIzol, and then the cDNA was obtained by reverse transcription using a reverse transcription kit (K1622; Thermo Fisher Scientific). qRT-PCR was performed using SYBR Green Master Mix (A25741, Thermo Fisher Scientific) to detect mRNA abundance. The primers used were as follows: *Tuj1*—F-5′-CATGGACAGTGTTCGGTCTG-3′, R-5′-CGCACGACATCTAGGACTGA-3′; *Map2*—F-5′-CTGGACATCAGCCTCACTCA-3′, R-5′-AATAGGTGCCCTGTGACCTG-3′; *Apol8*—F-5′-ATTGAGGAAGCCGCTGAGTA-3′, R-5′-CAGGTTTGCTGTGAGTGCAT-3′; and *Gapdh*—F-5′-TTCCATCCTCCAGAAACCAG-3′, R-5′-CCCTCGAACTAAGGGGAAAG-3′.

### Lentivirus production

The coding sequence of *Apol8* was inserted into the CD511B vector, which contains the GFP reporter gene, for lentivirus production in 293T cells. The concentration (titer) of lentivirus was 10^10^ TU/mL. The sequences of *Apol8* primers were F-5′-CCGGAATTCATGGACCCTTCAGACT-3′ (EcoRI site underlined), R-5′-CGATTTAAATGAACACCTCCCCGTC-3′ (SwaI site underlined). The lentivirus was used to infect NSCs during proliferation to enable *Apol8* expression before neuronal differentiation of NSCs.

### *Apol8*-NSC-LOCS graft preparation

The collagen nerve regeneration scaffold, LOCS, was soaked in poly-l-ornithine solution (P4957, Sigma) and then in fibronectin (F0895, Sigma) for 2 h at 37°C. After three washes, LOCS were cut to 2-mm lengths and placed in bundles of four to create a LOCS beam graft. Then *Apol8*-overexpression NSCs were digested to single cells and a million cells were attached to each LOCS beam. After incubation for 24 h, the graft was transplanted into the SCI injury site.

### Animal surgery and functional evaluation

Eight-week-old C57BL/6N mice were anesthetized by intraperitoneal injection of sodium pentobarbital (1%; 50 mg/kg), and then, a 1-mm section of the spinal cord was removed at the level of T8. When the fluid had ceased to ooze from the damaged area, the prepared graft was carefully placed in the damaged area. Finally, the muscle and skin were sutured. Penicillin was injected intraperitoneally for 1 week, and bladder massage was performed twice a day to assist urination. Motor function was evaluated weekly according to the Basso Mouse Scale (BMS) in a double-blind format [27]. Eight weeks after the surgery, the mice were anesthetized with 1% sodium pentobarbital, and the electrophysiological analyses were performed.

### Retrograde tracing using a pseudotyped rabies virus

A retrovirus was used to label proliferating NSCs [28], including normal NSCs and those treated with Epothilone D in vitro. The labeled NSCs were transplanted into the injured site. Seven weeks after SCI, a pseudotyped rabies virus (Rabies-EnvA-mCh) was injected into an undamaged site about 1 mm from the caudal end of the damaged area [29, 30]. Another week later, the mice were euthanized, and the spinal cord was obtained for histological analysis [17].

## Results

### Epothilone D promotes the neuronal differentiation of NSCs

**Fig. 1:**
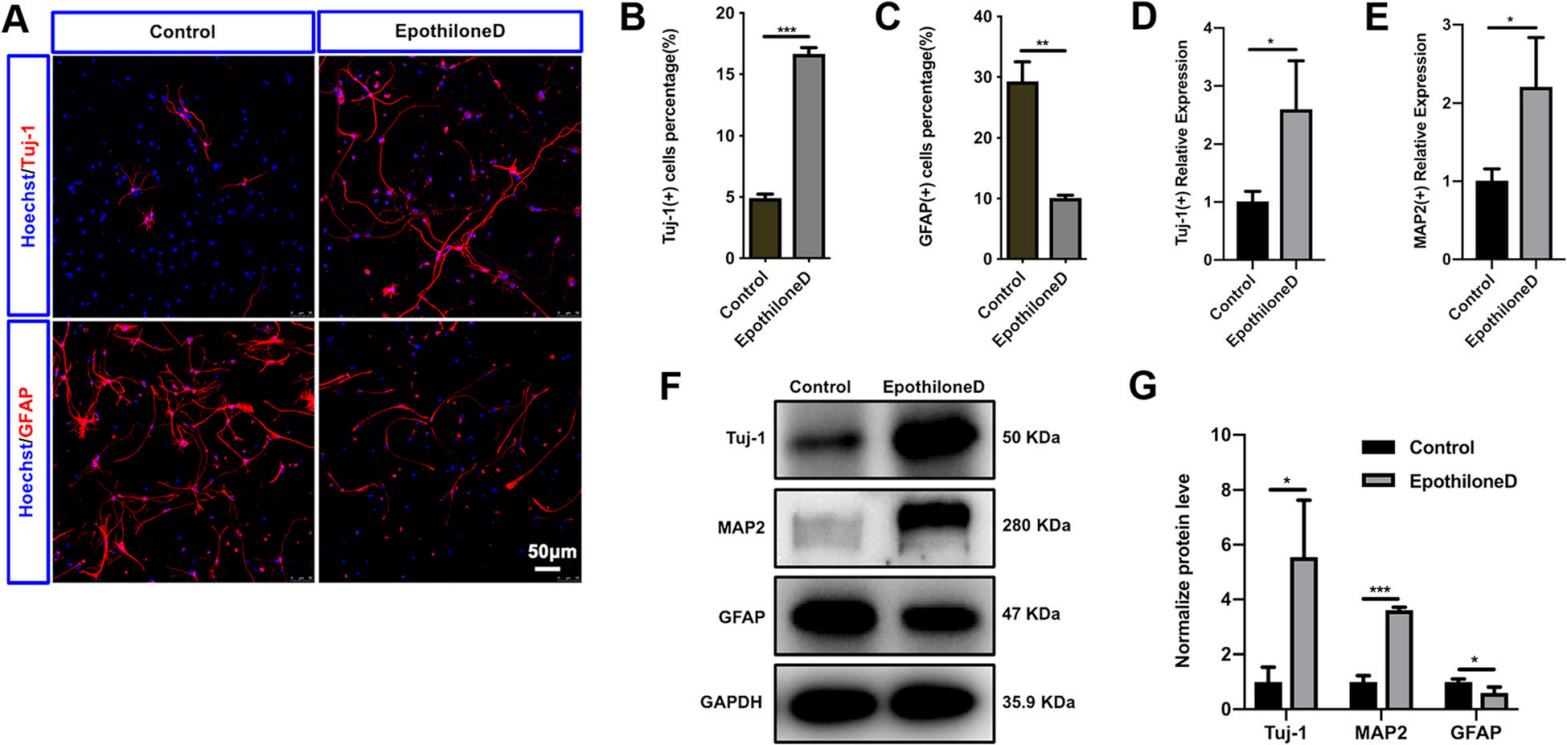
Epothilone D promotes NSC differentiation into neurons. **a** Epothilone D promoted the neuronal differentiation of spinal cord NSCs and inhibited their differentiation into astrocytes. Scale bars, 50 μm. **b** Quantification of percentages of TUJ1(+) cells among differentiated cells cultured with or without Epothilone D. **c** Quantification of percentages of GFAP(+) cells among differentiated cells cultured with or without Epothilone D. **d**, **e** *Tuj1* and *NeuN* mRNA levels in lysates of differentiated cells were analyzed by qRT-PCR after Epothilone D induction. **f**, **g** Western blot detection of the effect of Epothilone D on neuronal differentiation of NSCs. Error bars represent the mean ± SD

Epothilone D can stabilize microtubules [31] and can promote functional improvement of hind limb function in contusion spinal cord rats [32]. Some drugs that stabilize microtubules are used for SCI repair because they can promote NSC differentiation into neurons [20]; however, whether Epothilone D has this effect is unclear. We therefore explored the effect of Epothilone D on NSC differentiation. Under differentiation conditions, Epothilone D was added. On the 6th day, as shown in Fig. 1a, Epothilone D promoted NSC differentiation into neurons rather than astrocytes. The percentage of cells differentiating into neurons was promoted from 4.92 ± 0.73% in the control group to 16.68 ± 1.11% in the Epothilone D group, while the percentage of cells differentiating into astrocytes decreased from 29.33 ± 6.40% in the control group to 10.13 ± 0.83% in the Epothilone D group (Fig. 1a–c). The mRNA and protein levels of the neuronal markers TUJ1 and MAP2 were also detected on the 6th and 12th day of differentiation, respectively. The results showed that Epothilone D promoted mRNA expression of both TUJ1 and MAP2 (Fig. 1d, e). Western blot results were consistent with the expression levels of mRNAs (Fig. 1f, g). Together, these results demonstrated that Epothilone D promoted the differentiation of NSCs into neurons rather than astrocytes by inhibiting the proliferation of NSCs.

### Epothilone D can facilitate the reconstruction of neural circuits

**Fig. 2:**
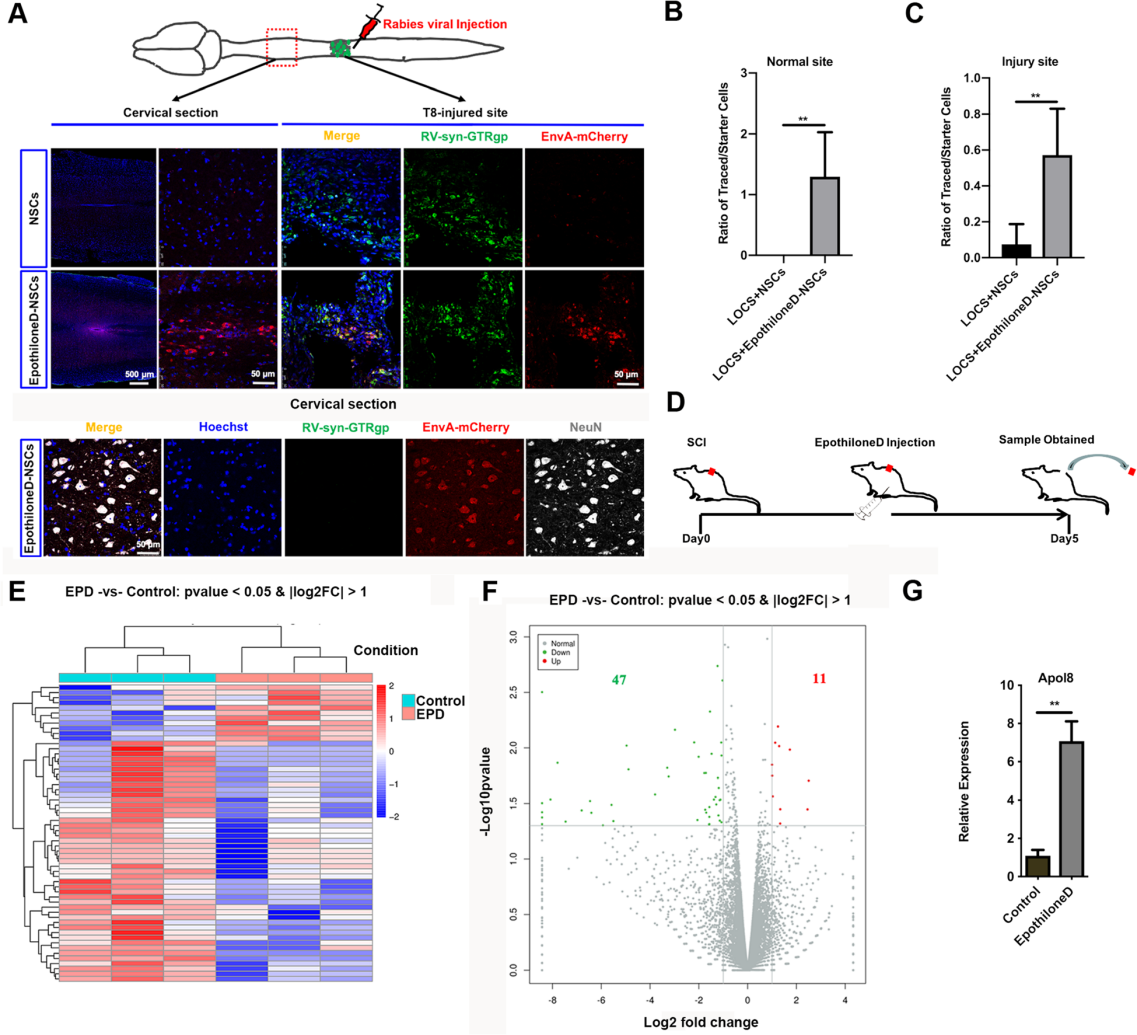
Transplantation of NSCs exposed to Epothilone D can promote neural circuit formation after SCI. **a** Pseudotyped rabies virus-mediated monosynaptic retrograde tracing was used to map the synaptic inputs to newborn neurons in the spinal cord. Transplantation of NSCs induced with Epothilone D promoted the formation of neural circuits in the injured site. Newborn cells (both green and red) in the injury site (T8) and traced cells (red only) in the normal spinal cord area (cervical section). Traced cells in the cervical section expressed NeuN protein. Scale bars are 500 μm or 50 μm. **b** The ratio of traced/starter cells was analyzed in the cervical section in **a**. **c** The ratio of traced/starter cells was analyzed in the injured site in **a**. **d** Diagram of the in vivo function patterns of Epothilone D before RNA-Seq. **e**, **f** RNA-Seq results revealed differentially expressed genes. **g** qRT-PCR analysis was used to detect upregulated differentially expressed gene during the differentiation of NSCs in vitro. Error bars represent the mean ± SD

Having shown that Epothilone D can promote the neuronal differentiation of NSCs in vitro, we then explored whether NSCs differentiated by Epothilone D could promote the reconstruction of neural circuits in the injured site after SCI. NSCs were stimulated with Epothilone D and were then labeled by infection with a retrovirus. Seven weeks after transplantation, pseudotyped rabies virus (Rabies-EnvA-mCh) was injected to trace the connectivity of newborn neurons in the spinal cord (mCh-positive cells represent traced cells and GFP/mCh double-positive cells represent newborn neurons). One week after virus injection, the ratio of new connectivity at the T8 injured site in the Epothilone D-NSC group was higher than that in the NSC group (Fig. 2a, c). However, the signal transmission was only detected in the Epothilone D-NSC group in the cervical region away from the damaged area (Fig. 2a, b). Immunofluorescence staining of NeuN showed that the signals transmitted by retrograde tracing technology were received by mature neurons in normal tissues; that is, functionally mature neurons in normal tissues were involved in the reconstruction of neural circuits with new mature neurons in damaged areas in the Epothilone D-NSC group (Fig. 2a).

To explore the mechanism by which Epothilone D promotes neuronal differentiation of NSCs, RNA-Seq was performed to determine differentially expressed genes in the control and Epothilone D addition groups. After SCI, Epothilone D was administered at the injured site, followed by daily intraperitoneal injections of Epothilone D for 5 days. Then, a 2-mm-long section of spinal cord tissue around the injury area was taken for RNA-Seq (Fig. 2d). Differentially expressed genes are displayed in the form of a heat map (Fig. 2e). Forty-seven genes were downregulated, and only 11 genes were upregulated (Fig. 2f). We speculate that some upregulated genes may be involved in the neuronal differentiation of NSCs by Epothilone D. qRT-PCR showed that *Apol8* was upregulated by Epothilone D during neuronal differentiation of NSCs in vitro (Fig. 2g). Therefore, we infer that Epothilone D may promote the neuronal differentiation that is involved in the reconstruction of neural circuits through *Apol8*.

### Epothilone D promotes neuronal differentiation of NSCs through *Apol8*

**Fig. 3:**
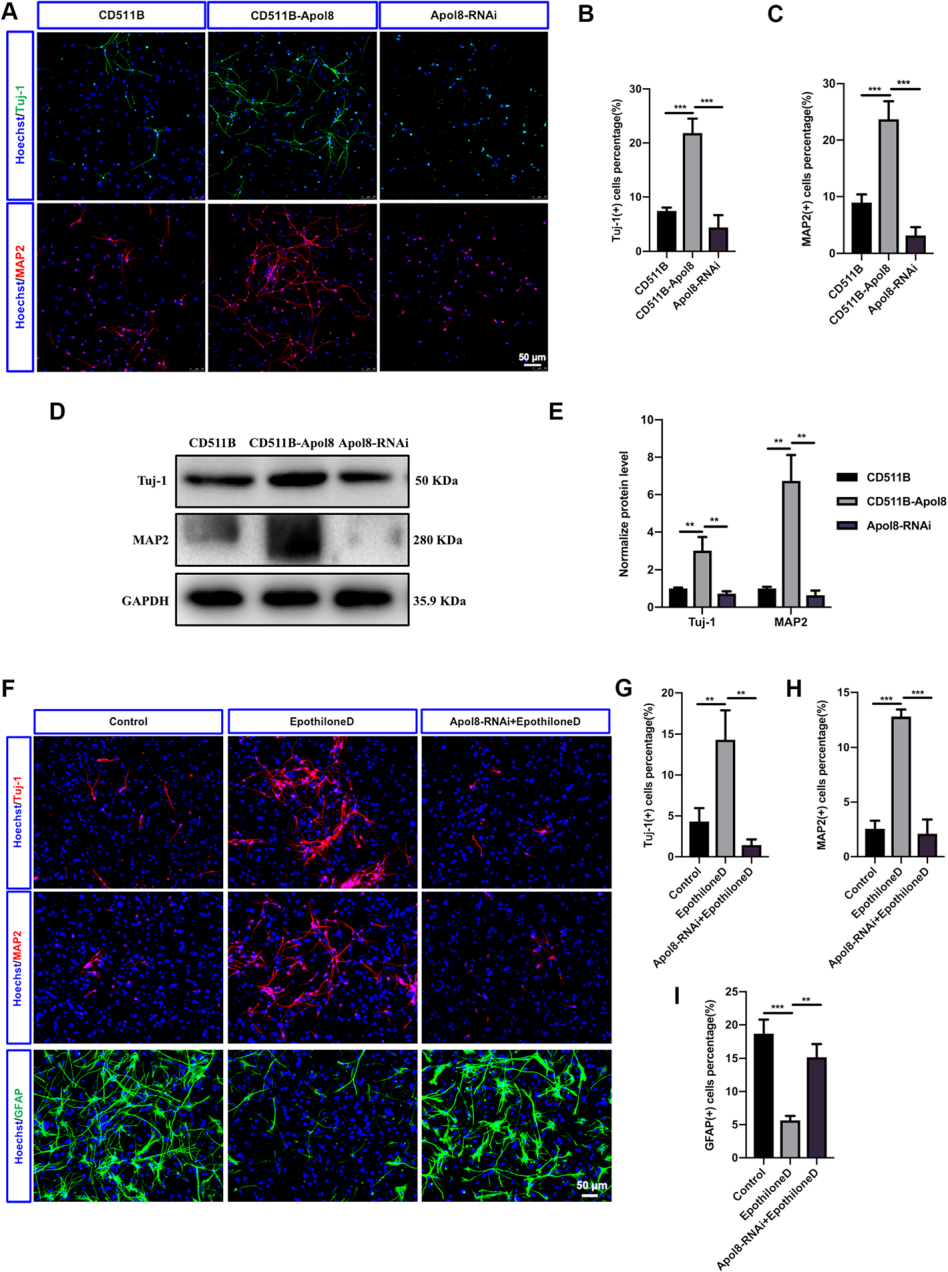
Epothilone D promotes neuronal differentiation of NSCs by upregulating *Apol8*. **a** Effects of *Apol8* on neuronal differentiation of NSCs through TUJ1 or MAP2 assessed by immunofluorescence staining. **b**, **c** Quantification of percentages of TUJ1(+) and MAP2(+) cells among differentiated cells cultured under *Apol8* overexpression or *Apol8*-RNAi conditions. **d**, **e** Western blotting was used to detect the effect of *Apol8* overexpression or *Apol8*-RNAi on neuronal differentiation of NSCs. **f** Effects of Epothilone D on neuronal differentiation of NSCs with or without the intervention of *Apol8*-RNAi, assessed by immunofluorescence staining. **g**–**i** Quantification of percentages of neurons or astrocytes in **F**. Error bars represent the mean ± SD. All scale bars, 50 μm

Next, we tested whether the upregulated differentially expressed genes were involved in the neuronal differentiation process of NSCs regulated by Epothilone D. Differentially expressed genes were inserted into the lentivirus overexpression vectors to explore their function, and overexpression of *Apol8* in NSCs was observed to promote neuronal differentiation of NSCs. The rate of neuronal differentiation was below 10% after 6 days of differentiation under spontaneous differentiation conditions. However, the neuronal differentiation rate was increased to 21.83 ± 2.68% by lentiviral overexpression of *Apol8* in NSCs. Conversely, the neuronal differentiation rate decreased to 4.40 ± 2.25% under *Apol8*-RNAi conditions (Fig. 3a, b). With a longer differentiation time (12 days), the percentage of MAP2(+) cells increased from 8.94 ± 1.45% in the control group to 23.72 ± 3.16% in the *Apol8* overexpression group and decreased to 3.13 ± 1.45% in the *Apol8*-RNAi group (Fig. 3a, c). The effect of *Apol8* on neuronal differentiation of NSCs was also shown to be evaluated by western blotting. Consistent with the immunofluorescence results, TUJ1 and MAP2 expression were improved by *Apol8* overexpression and inhibited by *Apol8*-RNAi during neuronal differentiation of NSCs (Fig. 3d, e). We therefore concluded that *Apol8* was involved in the neuronal differentiation of NSCs.

Next, we further explored whether Epothilone D can promote neuronal differentiation of NSCs through *Apol8* overexpression. We demonstrated that Epothilone D and *Apol8* can both promote neuronal differentiation; therefore, we designed an experiment in which *Apol8* was first knocked down by *Apol8*-RNAi technology, and then Epothilone D was used for induction. Epothilone D increased the percentage of TUJ1(+) neurons from 4.32 ± 1.64% in the control group to 14.32 ± 3.55%, and the percentage of mature neurons from 2.56 ± 0.74 to 12.81 ± 0.64% (Fig. 3f–h), while the percentage of astrocytes was decreased. Surprisingly, Epothilone D did not have a positive effect on neuronal differentiation following *Apol8* knockdown by *Apol8*-RNAi. As a result, the NSCs preferred to differentiate into astrocytes (Fig. 3f, i). In summary, *Apol8* was involved in the neuronal differentiation of NSCs induced by Epothilone D.

### NSCs overexpressing *Apol8* are more likely to differentiate into neurons after transplantation

**Fig. 4:**
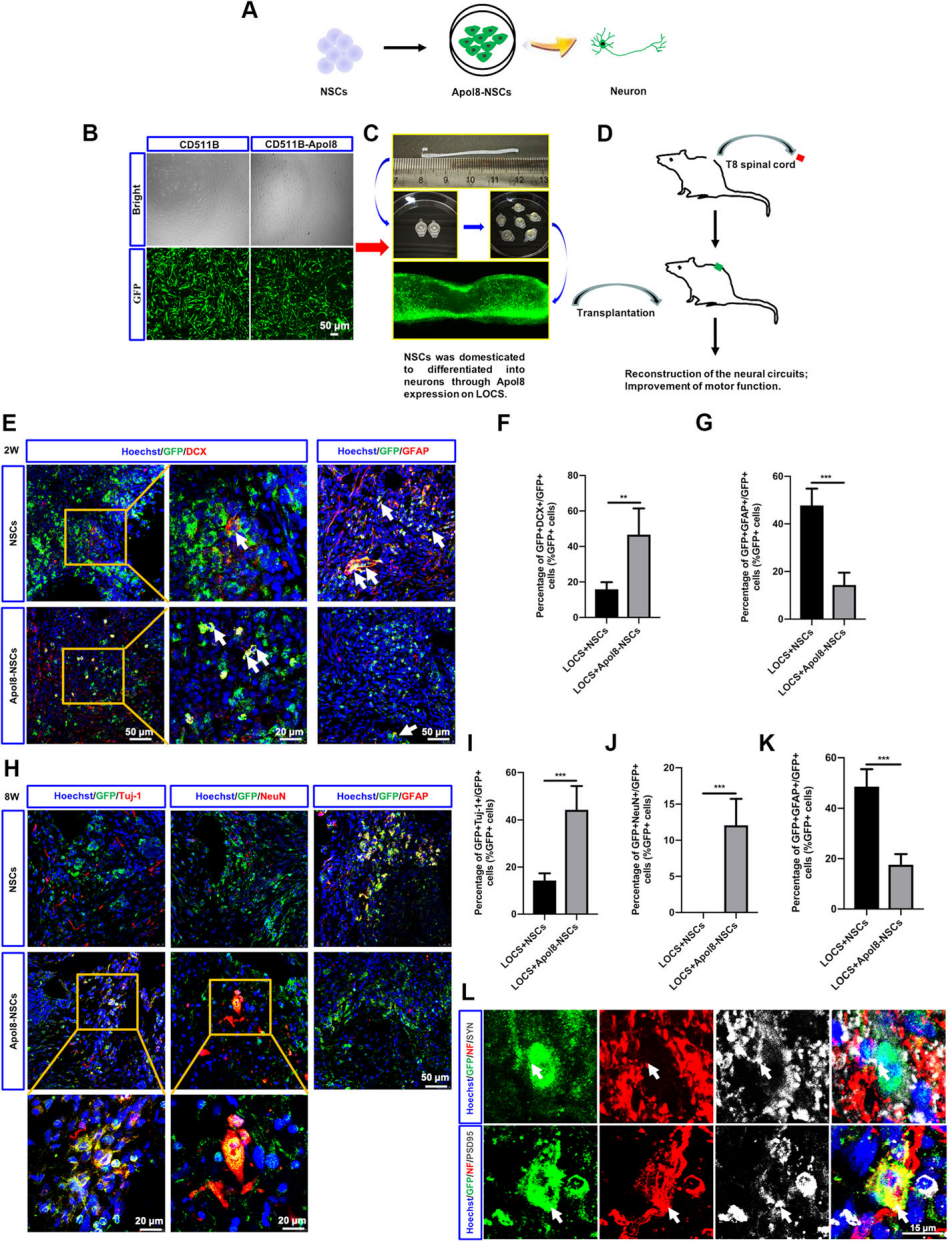
*Apol8* promotes neuronal differentiation of transplanted NSCs in vivo. **a** Patterns of neuronal differentiation of *Apol8*-NSCs. **b** *Apol8* overexpression in NSCs mediated by lentivirus. Scale bars, 50 μm. **c** NSCs with or without *Apol8* were loaded onto LOCS. **d** LOCS grafts loaded with NSCs were transplanted in the injury site after SCI. **e** Two weeks after transplantation, the spinal cord was dissected, and DCX and GFAP expression was determined by immunofluorescence staining. Scale bars are 50 μm or 20 μm. **f** Percentage of GFP(+)DCX(+)/GFP(+) cells in **e**. **g** Percentage of GFP(+)GFAP(+)/GFP(+) cells in **e**. **h** Eight weeks after transplantation, the spinal cord was dissected, and TUJ1, NeuN, and GFAP expression was determined by immunofluorescence staining. Scale bars are 50 μm or 20 μm. **i** Percentage of GFP(+)Tuj-1(+)/GFP(+) cells in **h**. **j** Percentage of GFP(+)NeuN(+)/GFP(+) cells in **h**. **k** Percentage of GFP(+)GFAP(+)/GFP(+) cells in **h**. **l** Transplantation of *Apol8*-NSCs on LOCS promotes the formation of synaptic structures in the injured site. Scale bars, 15 μm. Error bars represent the mean ± SD

After demonstrating that Epothilone D can promote the neuronal differentiation of NSCs by upregulating *Apol8*, we wondered whether the transplantation of NSCs overexpressing *Apol8* can overcome the inhibitory microenvironment by enhancing their intrinsic neuronal differentiation ability after SCI. First, lentivirus-mediated *Apol8* overexpression was used to replace Epothilone D-induced downstream *Apol8* expression, to endow NSCs with the potential to differentiate into neurons (Fig. 4a, b). Then, the NSCs were loaded onto LOCS and transplanted into the injured site after SCI (Fig. 4c, d). Two weeks later, NSCs tended to have differentiated into astrocytes rather than immature neurons (Fig. 4e). The percentage of GFP(+)DCX(+) transplanted NSCs was 15.89 ± 3.99%, and the percentage of GFP(+)GFAP(+) cells was as high as 47.75 ± 7.09% (Fig. 4f, g). However, the *Apol8*-NSCs loaded onto LOCS overcame the inhibitory SCI microenvironment and preferentially differentiated into neurons (46.68 ± 14.76%) rather than astrocytes (14.37 ± 5.16%) at the injury site (Fig. 4e–g). The proportion of early neurons was increased from about 15 to 46.68% ± 14.76% while the proportion of astrocytes was decreased from about 47 to 14.37 ± 5.16% (Fig. 4e–g). Next, the differentiation of NSCs was examined at 8 weeks after SCI. More *Apol8*-NSCs differentiated into neurons compared with non-transformed NSCs in the injured site (Fig. 4h). The percentage of TUJ1(+) cells was only 14.33 ± 3.00% in the NSC transplantation group but more than three times this in the *Apol8*-NSC group (Fig. 4i). At the same time, the proportion of astrocytes was still lower in the *Apol8*-NSC group (17.64 ± 4.20%) compared with that in the non-transformed NSC group (48.52 ± 6.89%) (Fig. 4k). Interestingly, *Apol8* also promoted the differentiation of mature neurons. Our results demonstrated that 12.08% of *Apol8*-NSCs differentiated into NeuN(+) neurons while no mature neurons were detected in the NSC transplantation group (Fig. 4j). We also detected synapse structures in the *Apol8*-NSC group through immunofluorescence staining for SYN and PSD95. The transplanted NSCs (GFP+) were surrounded by synaptic structures (NF+SYN+ or NF+PSD95+) (Fig. 4l). All of these results demonstrated that *Apol8* can help to improve the intrinsic neuronal differentiation ability of NSCs and promote the differentiation of transplanted NSCs into mature neurons by overcoming the inhibitory microenvironment after SCI, which contributes to the reconstruction of synaptic structures. This indicates that either Epothilone D-induced *Apol8* expression in vitro or lentivirus-mediated *Apol8* overexpression in vivo in NSCs can also promote neuronal differentiation and suggests that *Apol8* may play a similar role to Epothilone D, as a downstream molecule.

### *Apol8*-NSC transplantation enhances the recovery of motor function in mice with complete transection SCI

**Fig. 5:**
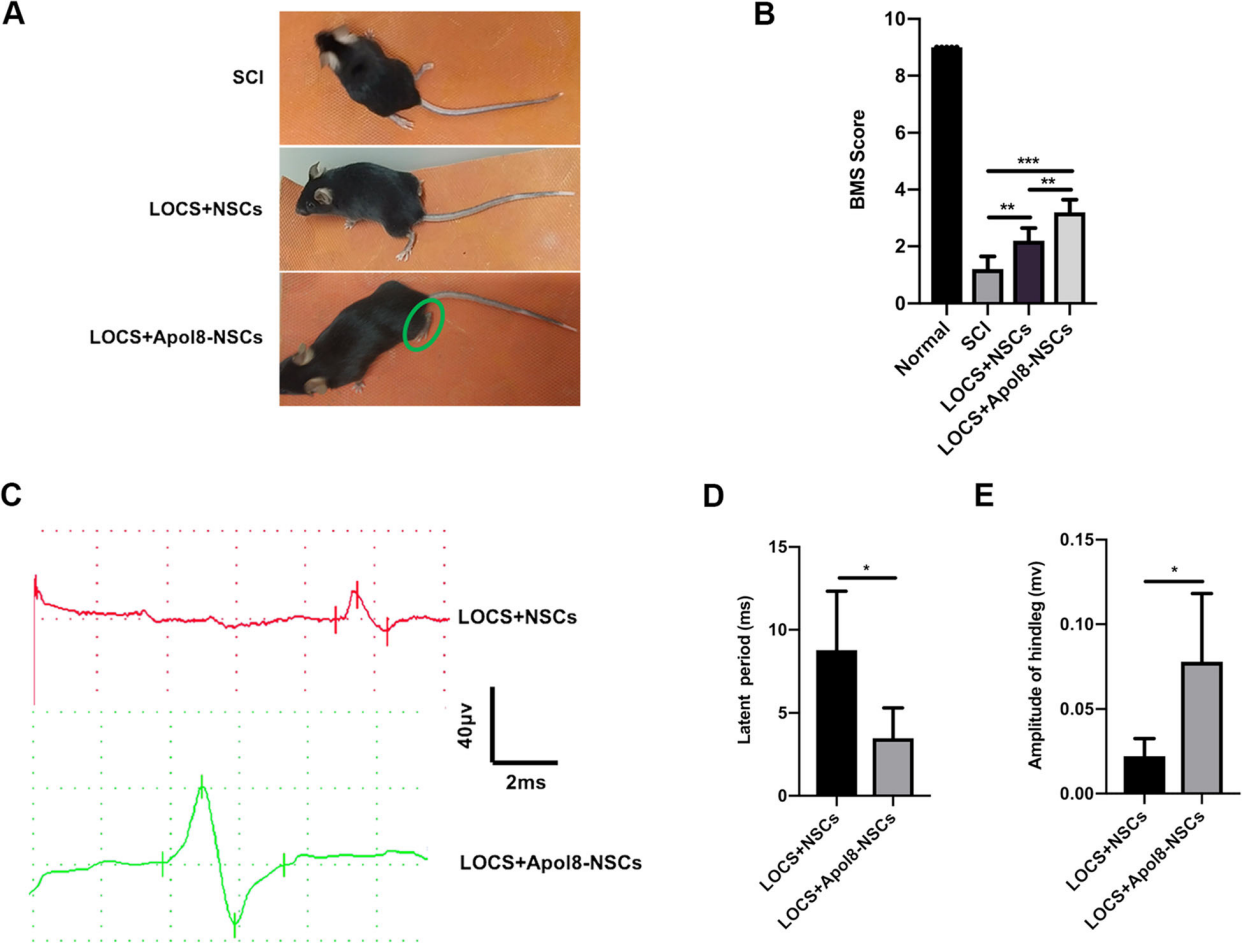
*Apol8*-NSC transplantation promotes improvement in motor function and electrophysiological recovery after SCI. **a** Representative functional recovery of animals in the SCI group, LOCS+NSCs group, and LOCS+*Apol8*-NSCs group at 8 weeks after SCI. **b** BMS scores of mice in each group at 8 weeks post-injury. **c** Motor evoked potential (MEP) results of LOCS+NSCs and LOCS+*Apol8*-NSCs treatment group mice at 8 weeks post-treatment. **d**, **e** Latent periods and amplitude ratio of MEP in LOCS+NSCs and LOCS+*Apol8*-NSCs treatment group mice, respectively. Error bars represent the mean ± SD

Epothilone D can help in functional recovery [32], and we have demonstrated that Epothilone D can facilitate the reconstruction of neural circuits through *Apol8*. We next tested whether the direct overexpression of Epothilone D-upregulated *Apol8* in NSCs can promote functional recovery in animals with SCI. The BMS was used to assess motor function after treatment for 8 weeks. The untreated mice showed slight ankle movements in their hind limbs (mean BMS score 1.2 ± 0.45). Both NSC and *Apol8*-NSC transplantation promoted functional recovery. However, mice occasionally stood on the balls of their feet in the *Apol8*-NSC transplantation group (mean BMS score 3.2 ± 0.45) while mice only achieved extensive ankle movement in the NSC transplantation group (mean BMS score 2.2 ± 0.45) (Fig. 5a, b). Electrophysiological results also showed that *Apol8*-NSC transplantation contributed to better electrophysiological recovery compared with NSC transplantation. *Apol8*-NSC-transplanted mice displayed a shorter latent period (3.48 ± 1.83 ms) and stronger amplitude (0.08 ± 0.04 mV) compared with mice in the NSC transplantation group (mean latent period 8.77 ± 3.55 ms; mean amplitude 0.02 ± 0.01 mV) (Fig. 5c–e). Our functional experiments showed that *Apol8*-NSC transplantation improved functional recovery after SCI, including motor function and electrophysiological function.

## Discussion

The inhibitory microenvironment of an injured site prevents transplanted NSCs from differentiating into mature neurons, which usually results in failed functional connections [33]. Previous studies have reported that Epothilone D can promote axon regrowth and facilitate functional recovery after SCI [34]. However, it remains unclear whether Epothilone D can contribute to SCI repair by promoting the neuronal differentiation of NSCs. In this study, we first found that Epothilone D can contribute to the neuronal differentiation of NSCs in vitro. Epothilone D can stabilize cell microtubules, which results in axon extension [35]. Furthermore, Epothilone D can inhibit cell proliferation through the regulation of cell cycle G1 checkpoint proteins [26]. The generation of neurons can be regulated by the cell cycle, and the distribution of genetic material determines the fate of cells through spindle microtubule stretching, which may be affected by some drugs that stabilize microtubules [16]. These findings indicate that the regulation of microtubules involved in the cell cycle can be affected by drugs such as Epothilone D, which may be a significant factor in the neuronal differentiation of NSCs; this is consistent with our present study. Our study points to a new strategy for screening neurogenic drugs and therapeutic drugs for SCI.

NSC transplantation has recently been used in SCI treatment [36]. However, directional differentiation is still a problem for SCI repair. The inhibitory microenvironment allows more NSCs to differentiate into astrocytes rather than neurons [6]. Gene-modified NSC therapies, in which fate determination of NSCs is regulated, are promising for SCI repair. Zeng et al. showed that NT3-overexpressing NSCs can promote TRKC-overexpressing NSCs to differentiate into neurons [37, 38]. Here, we demonstrated that Epothilone D can facilitate neuronal circuit construction in vivo and can promote the neuronal differentiation of NSCs through the downstream target, *Apol8*. We also demonstrated that overexpression of *Apol8* in NSCs can improve the percentage of neurons. To the best of our knowledge, this is the first time that *Apol8* has been shown to be involved in the neuronal differentiation of NSCs. Our previous study also showed that NSC transplantation with directional neuronal differentiation can promote functional recovery after SCI [39]. Therefore, in the present study, we attempted in vitro directional neuronal differentiation of NSCs by means of gene modification and then used these cells for SCI repair. Our results demonstrated improved neuronal differentiation of transplanted *Apol8*-NSCs compared with NSCs. The in vitro differentiation experiment also showed that the percentage of neuronal differentiation was higher in the *Apol8*-overexpression group compared with that in the Epothilone D group, which indicated that specific downstream target intervention plays a more important role in the fate determination of NSCs, which is significant for the treatment of SCI.

Understanding of the microenvironment after SCI is still very limited, and combination therapy has great potential for SCI repair. Biomaterials are widely used to improve repair after SCI [40]. Injectable and resorbable polypeptide hydrogels can enable axons to regrow across the SCI lesion site through the prolonged release of growth factors [41, 42]. We have previously reported that the collagen nerve regeneration scaffold, LOCS, has good biocompatibility and degradation properties and can guide the orderly growth of cells [43]. This indicated that LOCS can be used as an effective vector for delivering exogenous cells for cell transplantation therapy. We also demonstrated that transplantation of human mesenchymal stem cells and exogenous NSCs combined with LOCS can inhibit scar tissue formation, which can block nerve regrowth [44, 45]. Here, exploiting the good tissue compatibility of LOCS and genetic engineering technology, we overexpressed a target gene in NSCs after exploring the mechanism of Epothilone D function. Then, we delivered gene-modified NSCs combined with LOCS to the injury site. On the one hand, the SCI microenvironment was improved by LOCS, which guarantees the survival of the transplanted cells. On the other hand, the directional neuronal differentiation of NSCs involving genetic engineering makes directional neurogenesis possible and provides sources of cells for the reconstruction of neural circuits after SCI. Our results demonstrated that combination therapy promotes motor function recovery after complete SCI by combining LOCS and directed neuronal differentiation.

## Conclusions

This study demonstrated that Epothilone D promotes neuronal differentiation of NSCs by upregulating *Apol8*. The transplantation of NSCs overexpressing *Apol8* promoted the differentiation of NSCs into neurons and inhibited their differentiation into astrocytes in the injury site after SCI. Furthermore, differentiated neurons contributed to the reconstruction of neural circuits and the improvement of motor function after SCI, which is of significance for SCI repair.

## Data Availability

The data are available from the corresponding author on reasonable request.

## Abbreviations

BMS: Basso Mouse Scale
BSA: Bovine serum albumin
CNS: Central nervous system
DCX: Doublecortin
GAPDH: Glyceraldehyde 3-phosphate dehydrogenase
GFAP: Glial fibrillary acidic protein
GFP: Green fluorescence protein
LOCS: Linear ordered collagen scaffold
MAP 2: Microtubule-associated protein 2
NeuN: Neuronal N
NF: Neurofilaments
NSCs: Neural stem cells
PSD95: Postsynaptic density protein 95
qRT-PCR: Quantitative real-time polymerase chain reaction
RFP: Red fluorescent protein
RNA-Seq: RNA sequencing
RT: Room temperature
SCI: Spinal cord injury
SYN: Synaptophysin
TUJ1: Beta-tubulin III
